# Combining HIV prevention Options with Mental health service delivery for Adolescent girls and young women (CHOMA): results of a pilot hybrid effectiveness‐implementation randomized trial in South Africa

**DOI:** 10.1002/jia2.70037

**Published:** 2025-09-03

**Authors:** Jennifer Velloza, Nomhle Ndimande‐Khoza, Lisa Mills, Nicole Poovan, Aliza Adler, Elizabeth B. Sherwin, Carrie Mathew, Zinhle Sokhela, Ruth Verhey, Dixon Chibanda, Monica Gandhi, Connie Celum, Sinead Delany‐Moretlwe

**Affiliations:** ^1^ Department of Epidemiology & Biostatistics University of California San Francisco San Francisco California USA; ^2^ Department of Global Health University of Washington Seattle Washington USA; ^3^ Wits RHI, University of the Witwatersrand Johannesburg South Africa; ^4^ Friendship Bench Program Harare Zimbabwe; ^5^ Department of Medicine University of California San Francisco San Francisco California USA; ^6^ Department of Medicine University of Washington Seattle Washington USA; ^7^ Department of Epidemiology University of Washington Seattle Washington USA

**Keywords:** adherence, adolescent girls and young women, HIV, hybrid trial, mental health, pre‐exposure prophylaxis

## Abstract

**Introduction:**

Adolescent girls and young women (AGYW) at risk of HIV frequently have symptoms of common mental disorders (CMDs), which are associated with lower pre‐exposure prophylaxis (PrEP) adherence. We conducted a pilot hybrid effectiveness‐implementation trial (CHOMA) to evaluate whether an evidence‐based mental health intervention adapted for PrEP delivery (“Youth Friendship Bench SA”) could address CMD and PrEP adherence among South African AGYW.

**Methods:**

CHOMA was conducted in Johannesburg from April 2023 to February 2024. We enrolled AGYW (18−25 years) who were already on or willing to initiate PrEP and had CMD symptoms (Self‐Reporting Questionnaire 20‐item [SRQ‐20]≥7). Participants were randomized to our Youth Friendship Bench SA intervention (five problem‐solving sessions with a lay counsellor, one group session) or standard‐of‐care CMD services (brief CMD assessment, referral). Counselling sessions occurred at enrolment and Weeks 2, 4, 8 and 12. Co‐primary outcomes were PrEP adherence (positive result on a urine tenofovir assay) and reduced CMD symptoms (SRQ‐20<7) at Week 12 and, secondarily, Week 4. We used Poisson regression to assess intervention effects and summarized responses to three validated scales assessing intervention acceptability, appropriateness and feasibility (ranges: 1–4).

**Results:**

Of 116 AGYW enrolled, the median SRQ‐20 score was 9. We retained 69% through Week 12. Of 57 intervention participants, 64.9% (*N* = 37) received four or more sessions. At Week 4, 29/36 (80.6%) participants in the intervention and 25/41 (61.0%) in the standard‐of‐care had recent PrEP use (RR = 1.40; 95% CI = 1.03−1.89; *p* = 0.03), but this was not sustained through Week 12 (RR = 0.88; 95% CI = 0.64−1.22; *p* = 0.44). Enrolment SRQ‐20 score was not associated with Week 12 PrEP adherence or retention. CMD symptoms did not differ by arm at Week 4 or 12, although the proportion with SRQ‐20 scores >7 decreased overall between Weeks 4 (54.5%, 42/77) and 12 (35.0%, 28/80; *p* = 0.02). Median acceptability, appropriateness and feasibility scores were 3.50, 3.75 and 3.25, respectively.

**Conclusions:**

The intervention improved PrEP adherence at Week 4, although the effect was not durable to Week 12, possibly due to retention challenges. Reductions in CMD symptoms were seen in both arms. Findings suggest different mental health and PrEP support interventions may be needed to improve integrated service delivery among AGYW.

## 1 INTRODUCTION

HIV incidence is 4–7 per 100 person years among adolescent girls and young women (AGYW; ages 18–25) in some regions of Africa [1, 2, 3]. Pre‐exposure prophylaxis (PrEP) is a highly effective HIV prevention approach; however, AGYW have low rates of PrEP adherence partly due to symptoms of common mental disorders (CMDs; including depression, anxiety and stress) [4, 5, 6, 7, 8, 9, 10]. Adolescence is a typical period of CMD onset due to individual‐, interpersonal‐ and structural‐level factors, and 20–50% of AGYW seeking PrEP report CMD symptoms [8, 11]. South African AGYW with persistent CMD symptoms are 25% less likely to adhere to PrEP [7]. CMD symptoms may reduce PrEP adherence by affecting self‐efficacy, healthcare engagement and motivations to engage in protective healthcare behaviours [7]. Findings on associations between CMD risk and low PrEP adherence highlight the need to explore integrated interventions to address both HIV and CMD among AGYW in South Africa.

The standard‐of‐care (SOC) for addressing CMDs in South Africa involves a brief symptom screen in primary care, followed by referral as needed [12]. However, routine screenings are often not conducted, and referrals may not be made for AGYW who could benefit [13]. Integrating point‐of‐care psychotherapy may be a more feasible approach to improve CMDs among AGYW. Problem‐solving therapy (PST) is a psychotherapy that identifies actionable solutions to modifiable issues affecting one's mental health and improves coping around less modifiable issues [14, 15, 16]. The Friendship Bench is a PST intervention that has been effective at reducing mild‐to‐moderate CMDs among adults in similar African contexts [17]. It includes CMD screening, 4–6 individual problem‐solving sessions with a lay counsellor and group sessions on economic empowerment [17]. Integrating evidence‐based psychotherapy, like the Friendship Bench, into HIV service delivery has the potential to improve CMD and PrEP‐related outcomes for AGYW [18].

We previously conducted a four‐phase human‐centred design process with AGYW and key stakeholders to adapt the Friendship Bench to the needs of South African AGYW seeking PrEP in public clinics (the resulting intervention is called “Youth Friendship Bench SA”) [19]. This study sought to pilot the effectiveness and implementation of the Youth Friendship Bench SA, integrated with PrEP delivery, among South African AGYW.

## 2 METHODS

### 2.1 Study design and setting

The “Combining HIV prevention Options with Mental health service delivery for Adolescents” (CHOMA) effectiveness‐implementation hybrid type 1 study was a randomized pilot trial comparing the Youth Friendship Bench SA intervention to South African SOC for CMDs in primary care [19, 20]. This pilot was conducted at the Gauteng Ward 21 Adolescent Clinic (a public clinic offering PrEP to AGYW) and the Wits RHI Ward 21 Clinical Research Site in Johannesburg from April 2023 to February 2024. The protocol was registered at ClinicalTrials.gov on 15 December 2022 (identifier NCT05664490). Study information is reported in accordance with CONSORT guidelines [21, 22].

### 2.2 Participants

Participants were recruited through outreach at primary care facilities, social media and word‐of‐mouth from April 2023 to October 2023. Eligible participants were female, 18–25 years, HIV negative, taking PrEP (based on clinic records) or interested in initiating PrEP at enrolment and had a score ≥7 (indicating at least mild CMD symptoms) on the Self‐Reporting Questionnaire 20‐item (SRQ‐20), a validated CMD screening tool used in other Friendship Bench studies [23, 24, 25, 26, 27]. Potential participants could receive PrEP from either the Gauteng Ward 21 Clinic or the Ward 21 Clinical Research Site to streamline our mental health services with PrEP delivery. AGYW were excluded if they reported suicidal intent or self‐harm or had an active, unmanaged psychiatric condition; both groups were referred to a mental health provider.

### 2.3 Randomization and masking

Participants were assigned (1:1) to either the Youth Friendship Bench SA intervention *plus* SOC CMD services or SOC alone, using REDCap. The allocation sequence was generated using random numbers with variable block sizes ≤10 (R Core Team, 2022). Members of the local study team consented and randomized AGYW. Investigators were blinded to assignments.

### 2.4 Intervention package

The Youth Friendship Bench SA intervention included five individual counselling sessions at enrolment, Week 2, Week 4, Week 8 and Week 12, with the option for Week 2 and 8 sessions to be in person or via phone. Sessions were led by trained lay counsellors. Prior to the study launch, the Principal Investigator (JV) received a week‐long Friendship Bench training by one of its developers (RV). JV then conducted a 2‐week training with the two lay counsellors on the Youth Friendship Bench SA intervention, CMD presentations among AGYW and PST skills. The supervision team included a research psychologist (LM) who met with counsellors regularly to discuss cases. Sessions were audio‐recorded and reviewed by the study research psychologist and lead counsellor.

Counsellors led intervention participants through six steps of the Youth Friendship Bench SA: “opening up the mind” (talking about challenges affecting mental health and/or PrEP use), setting goals, defining a top problem, brainstorming solutions, selecting solutions and developing an action plan. During the enrolment session, the counsellor typically conducted all steps; this session was often the longest (∼60 minutes). During subsequent sessions (20−30 minutes), counsellors reviewed the participant's action plan and discussed any challenges they had in implementing it and any other recent mental health concerns.

The intervention also included a remote group counselling session (∼120 minutes) focused on economic empowerment. The session was offered between Weeks 8 and 12 via Zoom. It included content around preparing for school applications and job interviews. All counselling sessions were conducted in English or isiZulu.

### 2.5 Standard‐of‐care

SOC services followed the South African Adult Primary Care Guidelines [12], which include a brief CMD screening [28], followed by referral for mental healthcare assessment by a social worker, psychologist or doctor. We enhanced SOC services by incorporating protocolized CMD symptom assessment (with the Patient Health Questionnaire [PHQ]‐2 and SRQ‐20) at enrolment, Week 4 and Week 12, offering brief discussions with staff on risk of self‐harm and providing follow‐up on referrals to create a “warm hand‐off” for linkage with further care.

SOC for all participants included routine PrEP delivery procedures. PrEP refills were offered by clinics 1 month after PrEP initiation and quarterly thereafter.

### 2.6 Procedures

Following eligibility assessment, participants completed interviewer‐administered questionnaires on: depressive symptoms (9‐item Patient Health Questionnaire [PHQ‐9]) [29]; anxiety symptoms (Generalized Anxiety Disorder 7‐item [GAD‐7]) [30]; post‐traumatic stress (Primary Care PTSD Screen [PC‐PTSD]) [31]; alcohol use (Cut, Annoyed, Guilty, and Eye) [32]; gender‐based violence ([GBV]) [33]; coping (Coping Self Efficacy scale) [34]; self‐esteem (Rosenburg Self‐Esteem scale) [35]; sexual relationship power [36]; and sexual behaviour [37, 38]. Follow‐up visits with repeat data collection occurred at Weeks 4 and 12 (all participants). Intervention participants attended in‐person or remote counselling visits at Weeks 2 and 8.

Urine samples were collected from all participants at Weeks 4 and 12 and were tested for tenofovir (TFV) using a point‐of‐care assay that assesses PrEP dosing in the last 4–7 days [39, 40, 41]. The result is available within 2–3 minutes and was used to determine PrEP adherence and provide motivational adherence counselling to all participants at Weeks 4 and 12 [42, 43]. Those with detectable TFV were encouraged to continue PrEP. Those with undetectable TFV were asked how they could be supported. Motivational counselling was performed by the same counsellors who conducted the Youth Friendship Bench SA—for intervention participants, it was done as part of CMD counselling sessions.

At Week 12, intervention participants answered interviewer‐administered, Likert‐style questions on intervention acceptability (Acceptability of Intervention Measure [AIM]), appropriateness (Intervention Appropriateness Measure [IAM]) and feasibility (Feasibility of Intervention Measure [FIM]) [44].

### 2.7 Outcomes

The co‐primary effectiveness outcomes were reduced CMD symptoms, measured as an SRQ‐20 score <7 at Week 12, and PrEP adherence, measured as a detectable TFV on the urine assay at Week 12 [23]. As secondary outcomes, we explored shorter‐term intervention effects on reduced CMD symptoms and PrEP adherence at Week 4 and on continuous SRQ‐20, PHQ‐9 and GAD‐7 scores over time. We also assessed the intervention's effect on a composite, binary outcome of both SRQ‐20<7 and a positive urine assay result. Implementation outcomes included median AIM, IAM and FIM scores.

### 2.8 Adverse events

Serious adverse events (SAEs) were those that result in death, hospitalization and/or disability. We recorded reports of self‐harm, which were evaluated by clinicians to ensure appropriate action plans.

### 2.9 Sample size

The study had 80% power to detect a 20% difference in PrEP adherence between the two arms among 110 AGYW, assuming 40% of participants in the SOC arm were adherent to PrEP as seen in prior studies and 10% missing data at Week 12 [37, 45]. We also had 80% power to detect a 25% difference in the proportion of participants with reduced symptoms of CMD between arms, assuming 50% prevalence of CMD symptoms (as seen in prior studies [37, 45]) and 10% missing data at Week 12.

### 2.10 Data analysis

We used descriptive statistics to depict SRQ‐20 scores and PrEP adherence. For our primary intent‐to‐treat (ITT) analyses, we used Poisson regression with a log‐link and robust standard errors to assess the effect of our intervention on reduced CMD symptoms and PrEP adherence at Week 12 (primary outcome) and Week 4 (secondary outcome). We used multiple imputation using chained equations (MICE) to account for missingness due to missed visits and early terminations [46]. Randomly imputed values were generated from 100 imputations, and to meet assumptions that data were conditionally missing at random, we included baseline variables associated with missingness in our imputation model [46].

We conducted a per‐protocol analysis with the subset who had CMD and urine point‐of‐care assay data at Week 12. We restricted our intervention sample to participants who received a “full dose” (4−5 counselling sessions). We repeated the per‐protocol analyses for CMD and PrEP adherence outcomes at Week 4. All the above analyses were pre‐specified in our Statistical Analysis Plan and approved by our DSMB.

We conducted post‐hoc secondary and exploratory analyses. First, we assessed variables associated with CMD symptoms and PrEP adherence at Week 12. We conducted univariable Poisson regressions, adjusted for arm, with variables selected *a priori* based on the literature. Any variables significantly associated with outcomes (using *p*<0.05) were included in multivariable models. Second, we examined the intervention effect on continuous SRQ‐20, PHQ‐9, GAD‐7 and PC‐PTSD scores. Third, we examined the effect of the intervention on our composite CMD and PrEP outcome at Weeks 4 and 12 with Poisson regression. To explore whether and why the Youth Friendship Bench SA intervention may have worked for some and not others, we analysed case study data from a purposive sample of intervention participants with the largest and smallest changes in their SRQ‐20 scores (see Supplementary Case Studies).

We created median AIM, IAM and FIM scale scores (range: 1−4). We used Wilcoxon rank sum tests to compare median scores by Week 12 PrEP adherence and CMD symptoms. All analyses were conducted using R Core Team (2020).

### 2.11 Ethics statement

The protocol was approved by ethics review boards at the University of the Witwatersrand and UCSF. Participants provided written informed consent prior to participation.

## 3 RESULTS

### 3.1 Participant characteristics and retention

**Figure 1:**
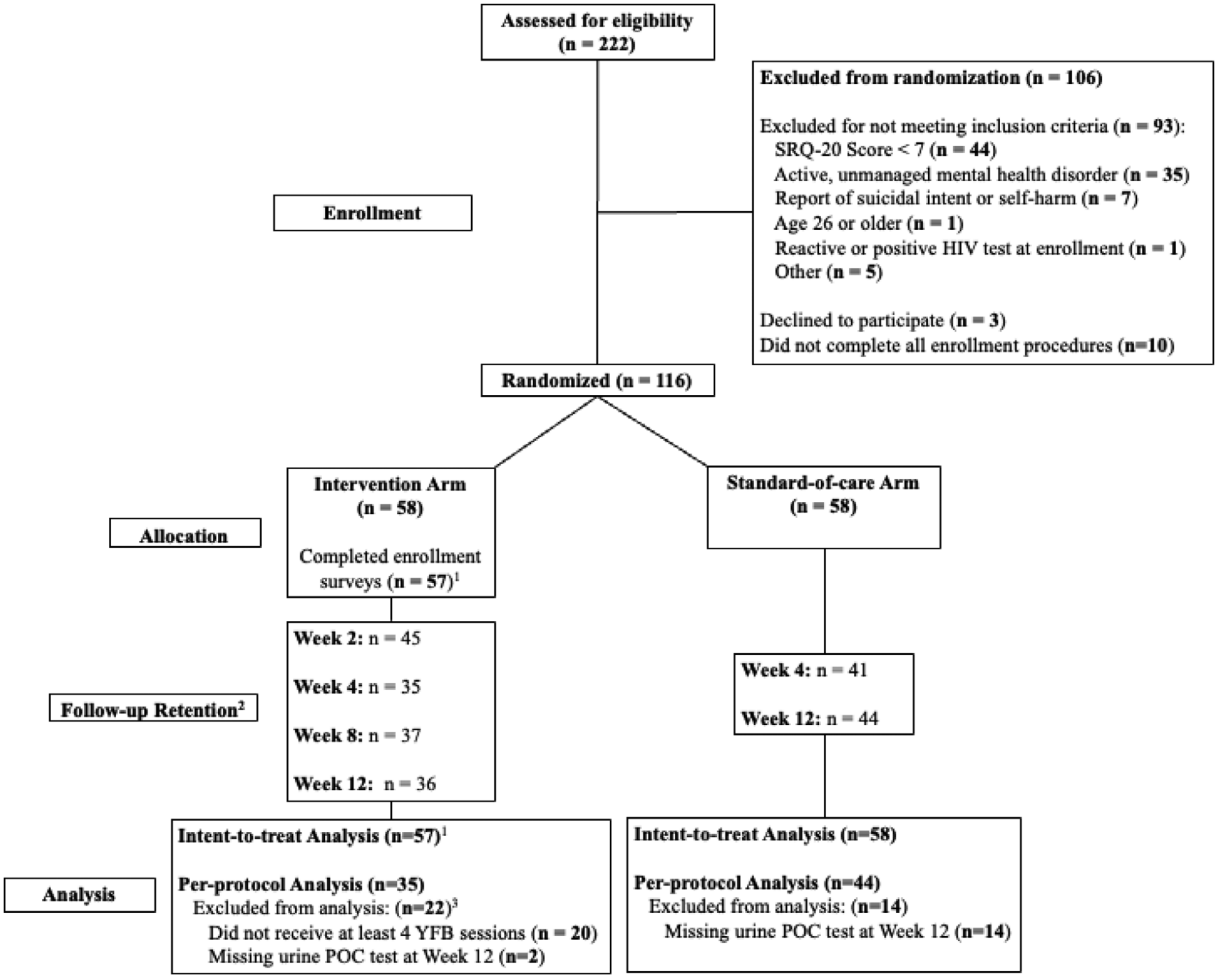
Participant flowchart diagram for the CHOMA study.

From April to October 2023, we enrolled 116 (52.3%) AGYW (Figure 1). Fifty‐eight (50%) were assigned to the Youth Friendship Bench SA plus SOC; however, one intervention participant did not complete enrolment procedures and was immediately removed from the study (*N* = 115 participants). At enrolment, the median SRQ‐20 score was 9 ([interquartile range] IQR = 7−10; Table 1). A total of 82 participants newly initiated PrEP at enrolment, and 33 initiated PrEP prior to enrolment. Of these 33 prior PrEP users, 24 (72.7%) had been using PrEP for less than 1 month at enrolment, three (9.1%) had been using PrEP for 1–3 months and six (18.2%) had been using PrEP for >3 months.

**Table 1:** Enrolment demographic, behavioural and mental health characteristics of CHOMA participants

	*N* (%) or median (IQR)^a^	
	Intervention *N =* 57^b^	Standard‐of‐care *N =* 58
***Demographics***		
Age, years	21.0 (19.0−22.0)	21.0 (20.0−23.0)
Relationship status		
Has romantic or sexual partner	45 (78.9%)	50 (86.2%)
Single, no partner	12 (21.1%)	8 (13.8%)
Highest level of education		
Primary school, complete	1 (1.8%)	0 (0.0%)
Some secondary school, not complete	9 (15.8%)	8 (13.8%)
Secondary school, complete	26 (45.6%)	31 (53.4%)
Some college or university, not complete	20 (35.1%)	15 (25.9%)
College or university, complete	1 (1.8%)	4 (6.9%)
Currently in school	26 (45.6%)	19 (32.8%)
Primary occupation		
Student	26 (45.6%)	18 (31.0%)
Unemployed	25 (43.9%)	29 (50.0%)
Employment with a steady salary	4 (7.0%)	5 (8.6%)
Employment without a steady salary	0 (0.0%)	2 (3.5%)
Parent caring for own children	2 (3.5%)	4 (6.9%)
Has a regular place to live	57 (100.0%)	58 (100.0%)
Living with^c^		
Alone	3 (5.3%)	2 (3.5%)
Partner	4 (7.0%)	5 (8.6%)
Parents	32 (56.1%)	37 (63.8%)
Siblings	28 (49.1%)	32 (55.2%)
Roommates(s)	5 (8.8%)	1 (1.7%)
***Mental health***		
SRQ‐20^d^ score	9.0 (8.0−10.0)	8.0 (7.0−10.0)
PHQ‐9^e^ score	8.0 (5.0−12.0)	8.5 (6.3−11.0)
Anxiety symptoms^f^	7.0 (5.0−11.0)	6.0 (4.3−10.0)
Positive post‐traumatic stress screen^g^	28 (49.1%)	20 (34.5%)
Positive alcohol or drug use screen^h^	19 (33.3%)	23 (39.7%)
Previous mental health diagnosis^i^	4 (7.0%)	2 (3.5%)
Current treatment for mental health diagnosis^i^	0 (0.0%)	1 (1.72%)
Previous treatment for mental health diagnosis^i^	1 (1.8%)	4 (6.9%)
Ever experienced any physical, sexual or emotional gender‐based violence^j^	44 (77.2%)	41 (70.7%)
Coping self‐efficacy score^k^	19.0 (16.0−23.0)	18.0 (15.0−23.0)
Self‐esteem score^l^	27.0 (24.0−31.0)	27.0 (23.2−30.0)
***Sexual behaviour***		
Total sexual partners in the last month^m^	1.0 (1.0−2.0)	1.0 (1.0−2.0)
Think sex partner has had sex with anyone else in the last month^n^		
Yes	13 (22.8%)	14 (25.0%)
No	7 (12.3%)	9 (16.1%)
Don't know	34 (59.6%)	28 (50.0%)
N/A (no partner in the last month)	3 (5.3%)	5 (8.9%)
Number of vaginal sex in the last month^o^	2.0 (1.0−4.0)	3.0 (2.0−4.0)
Condom use during vaginal sex in the past month^p^		
Never	10 (18.2%)	11 (19.3%)
Rarely	5 (9.1%)	8 (14.0%)
Sometimes	18 (32.7%)	20 (35.1%)
Often	5 (9.1%)	5 (8.8%)
Always	12 (21.8%)	7 (12.3%)
N/A (no vaginal sex in the last month)	5 (9.1%)	6 (10.5%)
Had transactional sex in the last month^q^	11 (19.3%)	11 (19.0%)
Sexual relationship power score^r^	12.0 (11.0−15.0)	12.0 (10.0−13.0)

Abbreviations: CHOMA, “Combining HIV prevention Options with Mental health service delivery for Adolescents”; IQR, interquartile range; PHQ‐9, Patient Health Questionnaire 9‐item; SRQ‐20, Self‐Reporting Questionnaire 20‐item.

^a^ Data are presented as median (interquartile range) for continuous variables and frequency (percentage) for categorical variables.

^b^ Fifty‐eight randomized to intervention but only 57 completed demographic information.

^c^ Total is greater than 100%, as participants can endorse multiple categories.

^d^ Possible range 0–20. A higher score indicates more depressive symptoms.

^e^ Possible range 0–27. A higher score indicates more depressive symptoms.

^f^ Anxiety symptoms assessed by Generalized Anxiety Disorder 7‐item scale (GAD‐7). Possible range 0–21. A higher score indicates more anxiety symptoms.

^g^ Post‐traumatic stress assessed by Primary Care PTSD Screen for DSM (PC‐PTSD) 4 item checklist. Positive screen if at least 3 items are endorsed.

^h^ Alcohol and drug use assessed by Cut Down, Annoyed, Guity, and Eye‐opener survey (CAGE) 4 items. Positive screen if at least 2 items are endorsed.

^i^ Mental health diagnoses include depression, anxiety, post‐traumatic stress and/or alcohol or drug use disorder.

^j^ Questions based on World Health Organization screening tool.

^k^ Assessed by Coping Self Efficacy (CSE) Scale. Possible range 0–39. A higher score indicates better coping self‐efficacy.

^l^ Assessed by Rosenburg Self‐Esteem Scale. Possible range 10–40. A higher score indicates higher self‐esteem.

^m^ Twelve missing values.

^n^ Two missing values.

^o^ Seventeen missing values.

^p^ Three missing values.

^q^ Includes sex in exchange for food, clothes/accessories/cosmetics, cell phone, items for children or family, transport, own school or residence fees, somewhere to stay or cash.

^r^ Assessed by Sexual Relationship Power Scale. Possible range 4–16. A higher score indicates higher sexual relationship power. Question was only asked to those who reported having at least one current sexual or romantic partner in the prior month. Data were available for *n* = 91 participants.

Visit completion was 66% at Week 4 and 69% at Week 12, and did not differ by arm (60.3% of intervention participants attended the Week 4 visit vs. 70.7% of SOC, *p* = 0.24; 62.1% of intervention participants attended the Week 12 visit vs. 75.9% of SOC, *p* = 0.11). Age (odds ratio [OR] = 0.81; 95% confidence interval [95% CI]: 0.64−0.99; *p*‐value = 0.05) and coping self‐efficacy (OR = 1.09; 95% CI: 1.01−1.19; *p*‐value = 0.03) were associated with missed visits and included in imputation models.

Of 57 intervention participants, 64.9% (*N* = 37) received the “full dose,” defined as four or more sessions, three (5.3%) attended three sessions, nine (15.8%) attended two sessions and eight (14.0%) attended one counselling session (at enrolment). Most intervention participants chose to have counselling via phone at Weeks 2 (*N* = 37; 82.2%) and 8 (*N* = 30; 81.1%).

### 3.2 Effectiveness outcomes

We included 115 participants in our ITT analyses. For our per‐protocol analyses (*N* = 79), intervention participants were excluded due to not receiving the full dose (*n* = 20) or missing PrEP adherence and/or CMD symptom assessment (*n* = 2), and 14 SOC participants were excluded due to missing PrEP and/or CMD data.

#### 3.2.1 CMD symptom reduction

**Figure 2:**
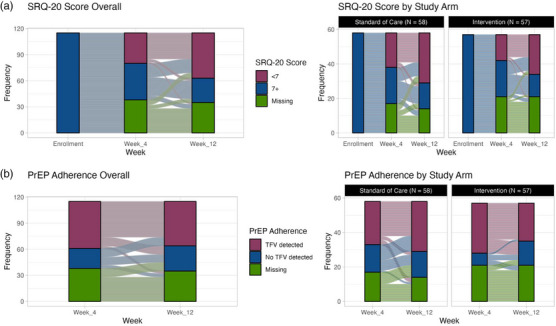
Alluvial plots depicting changes in SRQ‐20 scores and PrEP adherence throughout study period, overall and by arm (*N* = 115). PrEP, pre‐exposure prophylaxis; SRQ‐20, Self‐Reporting Questionnaire 20‐item.

CMD symptoms reduced significantly in the cohort over time (Figure 2A and Figure S1). At Week 12, 22.8% of 57 intervention participants had SRQ‐20 scores >7, compared with 25.9% of 58 in the SOC (relative risk [RR] = 1.00; 95% CI: 0.71−1.42; *p*‐value = 0.99; Table 2). Results were similar when per‐protocol analysis was performed (RR = 0.95; 95% CI: 0.68−1.33; *p*‐value = 0.78; Table 2). The Supplementary Case Studies provide narrative results to add context to these quantitative findings.

**Table 2:** Symptoms of common mental disorders at Weeks 4 and 12 by randomized arm

	Week 4 visit: Secondary outcome				Week 12 visit: Primary outcome			
Symptoms of common mental disorder^a^	Youth Friendship Bench SA	Standard‐of‐care	RR (95% CI)^b^	*p*‐value	Youth Friendship Bench SA	Standard‐of‐care	RR (95% CI)^b^	*p*‐value
**Intent‐to‐treat analysis (*N* = 115 at Weeks 4 and 12)** ^c^								
SRQ‐20 <7	15/57 (26.3%)	20/58 (34.5%)	0.81 (0.50–1.29)	0.37	23/57 (40.4%)	29/58 (50.0%)	1.00 (0.71–1.42)	0.99
**Per‐protocol analysis (*N* = 77 at Week 4 and *N* = 79 at Week 12)** ^d^								
SRQ‐20 <7	15/36 (41.7%)	20/41 (48.8%)	0.85 (0.52–1.41)	0.53	22/35 (62.9%)	29/44 (65.9%)	0.95 (0.68–1.33)	0.78

Abbreviations: 95% CI, 95% confidence interval; RR, relative risk; SRQ‐20, Self‐Reporting Questionnaire‐20 item.

^a^ Symptoms of common mental disorders assessed using the SRQ‐20. A score less than 7 indicates no or mild symptoms.

^b^ The reference category for all relative risks presented is the standard‐of‐care group.

^c^ The intent‐to‐treat analysis included all randomized participants. Relative risks, 95% confidence intervals and *p*‐values were calculated using multiple imputation for missing outcome data. The multiple imputation model included age and score on the Coping Self‐Efficacy scale, which were both predictive of retention in univariate models. Pooled results are presented in the RR (95% CI) and *p*‐value columns, while the *N*s and percentages shown are from the observed data only.

^d^ The per‐protocol analysis included data from all participants who attended Week 4 and/or Week 12 visits. For Week 12 per protocol, we restricted the intervention arm sample to those who received a full dose of the intervention, defined as 4–5 individual counselling sessions.

At Week 4, 36.8% of 57 participants in the intervention arm had SRQ‐20 scores >7 compared with 36.2% of 58 in the SOC (RR = 0.81; 95% CI: 0.50−1.29; *p*‐value = 0.37; Table 2). We also did not detect significant effects of the intervention in our per‐protocol (Table 2) or secondary analyses of change in continuous mental health scale scores (Tables S1–S3).

In multivariable Poisson regression models, only the PHQ‐9 score was associated with reduced CMD symptoms in the multivariable model (adjusted RR [aRR] = 0.95; 95% CI: 0.91−1.00; *p*‐value = 0.04). We provided referrals for additional CMD services to 39 participants in the SOC arm and 27 participants in the intervention arm.

#### 3.2.2 PrEP adherence

PrEP adherence remained stable over follow‐up in the cohort, with TFV detected in urine samples among 47.0% (*N* = 54) of participants at Week 4 and 44.3% (*N* = 51) of participants at Week 12 (Figure 2B).

At Week 12, 38.6% of 57 participants in the intervention arm had a positive urine assay (TFV was detected), compared with 50.0% of 58 in the SOC (relative risk [RR] = 0.88; 95% CI: 0.64−1.22; *p*‐value = 0.44; Table 3). At Week 4, 50.9% of 57 participants in the intervention arm had a positive assay compared with 43.1% of 58 in the SOC, indicating a significant effect of our intervention on short‐term PrEP adherence through 4 weeks (RR = 1.40; 95% CI: 1.03−1.89; *p*‐value = 0.03; Table 3). We did not identify any significant associations between demographic, mental health, and behavioural factors and PrEP adherence at Week 12 in multivariable models.

**Table 3:** PrEP adherence at Weeks 4 and 12 by randomized arm

	Week 4 visit: Secondary outcome				Week 12 visit: Primary outcome			
PrEP adherence^a^	Youth Friendship Bench SA	Standard‐of‐care	RR (95% CI)^b^	*p*‐value	Youth Friendship Bench SA	Standard‐of‐care	RR (95% CI)^b^	*p*‐value
**Intent‐to‐treat analysis (*N* = 115 at Weeks 4 and 12)** ^c^								
Positive urine TFV assay	**29/57 (50.9%)**	**25/58 (43.1%)**	**1.40 (1.03–1.89)**	**0.03**	22/57 (38.6%)	29/58 (50.0%)	0.88 (0.64–1.22)	0.44
**Per‐protocol analysis (*N* = 77 at Week 4 and *N* = 79 at Week 12)** ^d^								
Positive urine TFV assay	29/36 (80.6%)	25/41 (61.9%)	1.32 (0.99–1.77)	0.06	22/35 (62.9%)	29/44 (65.9%)	0.95 (0.68–1.33)	0.78

Bold indicates that a relative risk value was statistically significant, at the threshold of *p* < 0.05.

Abbreviations: 95% CI, 95% confidence interval; PrEP, pre‐exposure prophylaxis; RR, relative risk; TFV, tenofovir.

^a^ PrEP adherence assessed using the urine point‐of‐care assay. A positive assay result indicated PrEP use in the prior 4–7 days.

^b^ The reference category for all relative risks presented is the standard‐of‐care group.

^c^ The intent‐to‐treat analysis included all randomized participants. Relative risks, 95% confidence intervals and *p*‐values were calculated using multiple imputation for missing outcome data. The multiple imputation model included age and score on the Coping Self‐Efficacy scale, which were both predictive of retention in univariate models. Pooled results are presented in the RR (95% CI) and *p*‐value columns, while the *N*s and percentages shown are from the observed data only.

^d^ The per‐protocol analysis included data from all participants who attended Week 4 and/or Week 12 visits. For Week 12 per protocol, we restricted the intervention arm sample to those who received a full dose of the intervention, defined as 4–5 individual counselling sessions.

#### 3.2.3 Composite CMD symptom reduction and PrEP adherence outcome

At Week 12, 34.3% (*N* = 12/35) of participants in the intervention arm and 47.7% (*N* = 21/44) of participants in the SOC demonstrated a reduction in CMD symptoms and a positive TFV urine assay; this composite outcome was not different between arms (RR = 0.70; 95% CI: 0.40−1.22; *p*‐value = 0.21).

### 3.3 Implementation outcomes

Acceptability, appropriateness and feasibility scores were high among intervention participants at Week 12 (*N* = 35; Table 4). Over 90% of participants “strongly agreed” or “agreed” with all acceptability, appropriateness and feasibility statements. The median acceptability, appropriateness and feasibility scores were 3.50, 3.75 and 3.25, respectively. We found higher acceptability and feasibility scores for those with a positive urine TFV assay (median AIM and FIM scores of 3.75 each) than those with a negative result (median AIM and FIM scores of 3.25 and 3.00, respectively; *p*‐value = 0.04 for AIM and 0.05 for FIM comparison). There was no difference in IAM score by PrEP adherence or in comparisons of all three scores and CMD symptom reduction.

**Table 4:** Acceptability, appropriateness and feasibility of the Youth Friendship Bench SA intervention (among intervention participants only; *N* = 35)

Item	Strongly agree	Agree	Disagree	Strongly disagree	Median score (IQR)
**Acceptability** ^a^					
I approve of the Youth Friendship Bench SA	21 (60.0%)	13 (37.1%)	1 (2.9%)	0 (0.0%)	3.50 (3.0−4.0)
The Youth Friendship Bench SA is appealing to me	14 (40.0%)	19 (54.3%)	2 (5.7%)	0 (0.0%)	
I like the Youth Friendship Bench SA counselling sessions	19 (54.3%)	14 (40.0%)	2 (5.7%)	0 (0.0%)	
I welcome the Youth Friendship Bench SA counselling sessions	19 (54.3%)	13 (37.1%)	3 (8.6%)	0 (0.0%)	
**Appropriateness** ^b^					
The Youth Friendship Bench SA seems fitting for this clinic	20 (57.1%)	15 (42.9%)	0 (0.0%)	0 (0.0%)	3.75 (3.25−4.0)
The Youth Friendship Bench SA seems suitable for this clinic	22 (62.9%)	13 (37.1%)	0 (0.0%)	0 (0.0%)	
The Youth Friendship Bench SA seems applicable to the needs of young women	22 (62.9%)	12 (34.3%)	1 (2.9%)	0 (0.0%)	
The Youth Friendship Bench SA seems like a good match for the mental health needs of young women	23 (65.7%)	12 (34.3%)	0 (0.0%)	0 (0.0%)	
**Feasibility** ^c^					
The Youth Friendship Bench SA seems implementable	14 (40.0%)	19 (54.3%)	2 (5.7%)	0 (0.0%)	3.25 (3.0−4.0)
The Youth Friendship Bench SA seems possible for this clinic	15 (42.9%)	20 (57.1%)	0 (0.0%)	0 (0.0%)	
The Youth Friendship Bench SA seems doable for this clinic	15 (42.9%)	17 (48.6%)	3 (8.6%)	0 (0.0%)	
The Youth Friendship Bench SA seems easy to deliver to young women in PrEP settings	17 (48.6%)	15 (42.9%)	2 (5.7%)	1 (2.9%)	

Abbreviation: IQR, interquartile range.

^a^ Acceptability measured using the 4‐item Acceptability of Intervention Measure (AIM).

^b^ Appropriateness measured using the 4‐item Intervention Appropriateness Measure (IAM).

^c^ Feasibility measured using the 4‐item Feasibility of Intervention Measure (FIM).

### 3.4 Adverse events

Two SAEs were reported (neither related to the study). One intervention participant accidentally ingested rat poison (reported that she thought the poison was food). An SOC participant deliberately self‐harmed and was treated with hospital management and medications.

## 4 DISCUSSION

In this pilot effectiveness‐implementation study of the Youth Friendship Bench SA intervention in Johannesburg, CMD symptom reduction and PrEP adherence did not differ between our intervention and SOC arms at Week 12. We found a significant decrease in CMD symptoms in the cohort of AGYW, which may be due to more systematic implementation of SOC procedures than in real‐world settings. PrEP adherence was higher among intervention than SOC participants at Week 4, suggesting a possible short‐term effect of problem‐solving counselling on PrEP pill‐taking. PrEP adherence remained modestly high over time. The Youth Friendship Bench SA integrated with PrEP delivery was acceptable, feasible and appropriate.

While other studies have integrated mental health approaches with HIV testing and treatment interventions [47], ours is one of the first to incorporate CMD screening and evidence‐based psychotherapy within PrEP delivery. Several studies delivering problem‐solving and mindfulness counselling with HIV treatment in African settings have reported improvements in mental health symptoms, although these studies generally had short follow‐up times and some lacked a control group [48, 49, 50, 51, 52]. Findings on HIV outcomes from these studies were either mixed or not reported [48, 49, 50, 51, 52]. Only one integrated intervention found significant impacts on reduced depressive symptoms and improved HIV treatment adherence [53]; the effectiveness of integrated interventions on both mental health and HIV outcomes has generally not been demonstrated [54].

Hybrid effectiveness‐implementation approaches offer a method to explore clinical effectiveness of integrated packages alongside early implementation outcomes. The implementation outcomes may drive participants’ receipt of these interventions, willingness to meaningfully engage and health system factors that influence intervention success [20, 55, 56]. Two pilot effectiveness‐implementation trials incorporating PST (based off the Friendship Bench) with HIV treatment among adults in Zimbabwe and Malawi reported high acceptability, appropriateness and feasibility, and signals of improvements in CMD and HIV care outcomes, showing promise of PST when implemented well [25, 57]. While our findings add to this literature on integrated CMD and HIV services, AGYWs’ motivations for HIV prevention may be different than for treatment and there may be unique challenges to sustaining prevention behaviours in the context of more pressing psychosocial issues (e.g. it may be difficult to prioritize PrEP and clinic appointments for refills while feeling otherwise healthy and facing competing social or economic needs).

We did not find a difference in CMD and PrEP outcomes by arm, which could indicate a lack of effectiveness of our intervention. A high proportion of our sample had a positive screen for post‐traumatic stress and GBV, but the Friendship Bench may not have been the optimal intervention approach for AGYW with high exposure to sexual trauma and related anxiety. A Friendship Bench trial in Zimbabwe found that individuals with co‐morbid anxiety were more likely to have persistent CMD symptoms after receiving the intervention, and problem‐solving is limited among individuals with trauma histories [58, 59]. AGYW face a myriad of individual‐, interpersonal‐ and structural‐level challenges to mental health and PrEP adherence. While we hypothesized that our Youth Friendship Bench SA intervention could help a population with mild‐to‐moderate CMD symptoms problem solve around challenges within their control and improve coping behaviours around challenges outside of their control, our findings suggest that individual‐level counselling is likely insufficient to address their needs.

Our study offered encouraging results around CMD symptoms and PrEP adherence in the overall cohort. We implemented the SOC with trained lay counsellors, culturally appropriate instruments and warm handoffs to referrals. This was likely an enhancement over SOC, as it is conducted in public facilities and could explain why we saw CMD symptoms decline over time. Our findings suggest that, for many AGYW, SOC mental health services can effectively improve CMD symptoms when done routinely and in a youth‐friendly way. PrEP adherence remained high in both arms, possibly because the urine assay with motivational counselling assisted AGYW in taking pills regularly. Several other studies have also reported high, sustained levels of PrEP adherence among women who receive feedback from the urine assay [42, 60, 61].

Strengths included our hybrid effectiveness‐implementation design. We collected general (e.g. SRQ‐20) and disease‐specific (e.g. PHQ‐9) CMD measures. PrEP adherence was assessed via a urine TFV assay, which allowed for accurate, point‐of‐care assessment of recent pill‐taking. We offered intervention participants the option to conduct their Week 2 and 8 counselling via phone, which may have prevented further retention drop‐offs and offers an example of a hybrid in‐person/remote counselling model for AGYW.

This study also had several limitations. It was a pilot with a small sample and retention challenges (although retaining nearly 70% of participants was promising for AGYW with HIV risk compounded by CMD symptoms). Retention was lower in the intervention than in the SOC arm (albeit not significantly), which could indicate that participants struggled to return for scheduled sessions in the face of other challenges. We collected baseline survey data after randomization, which led to one participant being randomized to the intervention but not completing enrolment visit procedures. We reviewed recordings from a subset of counselling sessions to check fidelity to the counselling manual and support counsellors, but did not formally assess the quality of or fidelity to intervention delivery for in‐person or telephonic sessions. We did not collect data on PrEP adherence at enrolment because the majority of participants newly initiated PrEP; however, our PrEP analyses may be biased if baseline adherence differed by arm among participants who had been using PrEP prior to enrolment. While we offered referrals for additional CMD services to all participants, we did not track uptake of referral services. Finally, our results may not be generalizable in contexts where other social and structural factors drive HIV and CMD risk.

## 5 CONCLUSIONS

Reduction in CMD symptoms and objective PrEP adherence did not differ significantly among AGYW between SOC and intervention arms in a pilot trial when a PST intervention was used. There are several lessons learned from this study to inform integrated mental health and PrEP interventions. CMD symptom reductions and PrEP adherence estimates in the overall cohort are encouraging and suggest that “enhanced” SOC delivery with screening, warm handoffs and PrEP counselling with biomarker data may address mental health and PrEP adherence challenges for most AGYW. The Youth Friendship Bench SA may be appropriate for some individual‐ and interpersonal‐level problems, but is likely insufficient to address structural challenges and was not developed to combat severe CMD or PTSD symptoms. Multi‐level interventions that address structural factors, alongside long‐acting PrEP products, may be necessary to meaningfully improve mental health and HIV prevention for some AGYW. Future research is needed to evaluate optimal approaches to integrate mental health and PrEP interventions for AGYW in resource‐constrained settings.

## COMPETING INTERESTS

The authors report no conflicts of interest.

## AUTHOR CONTRIBUTIONS

JV, CC and SD‐M designed the study. JV, AA and EBS conducted all analyses. JV wrote the manuscript. All authors reviewed and approved the final version of the manuscript. JV: Funding, study design, data analysis, results interpretation, manuscript first draft. NN‐K: Study management, data collection, results interpretation, edited manuscript. LM: Study management, data collection, results interpretation, edited manuscript. NP: Study management, results interpretation, edited manuscript. AA: Data analysis, results interpretation, edited manuscript. EBS: Data analysis, results interpretation, edited manuscript. CM: Study management, results interpretation, edited manuscript. ZS: Data collection, edited manuscript. RV: Edited manuscript. DC: Edited manuscript. MG: Edited manuscript. CC: Study design, results interpretation, edited manuscript. SD‐M: Study design, study management, results interpretation, edited manuscript.

## FUNDING

This work was supported by award number R00 MH123369 (PI: Velloza) from the National Institutes of Mental Health (NIMH) of the National Institutes of Health (NIH). The urine tenofovir assays and MG were supported by 2R01AI143340 (PI Gandhi) from the National Institute of Allergy and Infectious Diseases (NIAID) of the NIH.

## DISCLAIMER

The content is solely the responsibility of the authors and does not necessarily represent the official views of the NIH.

## ETHICS APPROVAL STATEMENT

This study was performed in line with the principles of the Declaration of Helsinki. Approval was granted by the Ethics Committees of the University of California San Francisco (Date: 13 January 2023; IRB #22‐37680) and the University of the Witwatersrand (Date: 5 December 2022; IRB #221006).

## CONSENT STATEMENT

Written informed consent was obtained from all individual participants included in the study.

## CODE AVAILABILITY STATEMENT

Source code is available via CHOMA's GitHub repository; a link to the repository will be available upon reasonable request to the first author.

## Supporting information




**Figure S1**: Mean SRQ‐20 score over study period, by study arm (*N* = 79)
**Table S1**: PHQ‐9 score at Week 12 by randomized arm (*N* = 79)
**Table S2**: GAD‐7 score at Week 12 by randomized arm (*N* = 79)
**Table S3**: PC‐PTSD at Week 12 by randomized arm (*N* = 79)

## Data Availability

Data are available upon reasonable request to the first author.
