# Supplementary material for: Combining HIV prevention Options with Mental health service delivery for Adolescent girls and young women (CHOMA): results of a pilot hybrid effectiveness‐implementation randomized trial in South Africa
Source: J Int AIDS Soc. 2025 Sep 3;28(9):e70037. doi: 10.1002/jia2.70037 (PMC12408919; doi:10.1002/jia2.70037)
Supplement: Supplementary file 1 — Figure S1: Mean SRQ‐20 score over study period, by study arm (N = 79) Table S1: PHQ‐9 score at Week 12 by randomized arm (N = 79) Table S2: GAD‐7 score at Week 12 by randomized arm (N = 79) Table S3: PC‐PTSD at Week 12 by randomized arm (N = 79) [file JIA2-28-e70037-s001.docx]

**Supplemental Materials**

**Supplemental Case Studies**

***Methods:*** We purposively sampled 10% of intervention participants who attended at least 4 counseling sessions and had SRQ-20 data at Week 12. We stratified the population to select three participants who responded well to the intervention (defined as having at least a 5 point decrease in SRQ-20 score from Enrollment to Week 12, with a Week 12 SRQ-20 score <7) and three participants who seemed to not respond as well to the intervention (no decrease or an increase in SRQ-20 score from Enrollment to Week 12). We reviewed counseling session case report forms, which were completed by lay counselors following each session and included open-ended questions about the top problems selected, the participant’s action plan, and any challenges in conducting the action plan since the prior session. These case study data were summarized in narrative form to add context to our quantitative findings.

***Results:*** Among the three randomly selected participants who responded well to the intervention, all were 21-25 years and their presenting “top problems” included challenges with gaining employment (ID #252), interpersonal issues with family members and teachers (ID #235), and the death of a sibling (ID #272). Participants #252 and #235 developed action plans with their counselors focused on modifying these problems through developing job applications and plans for soliciting interviews (for 252) and practicing constructive conversations to discuss interpersonal challenges with others (for 235). Both reported being able to complete their action plans within the first two Youth Friendship Bench SA sessions. Participant 272 developed an action plan around opening up to other people to help with her coping and reported that just talking to the counselor helped improve her CMD symptoms.

Among the three participants who did not respond well to the intervention or have a reduction in the CMD symptoms, all were 18-20 years, all had ever experienced GBV, and their presenting “top problems” included financial and housing insecurity (#271, #288) and concerns about a child who had been sexually assaulted (#263). These participants reported being unable to secure housing or find jobs during the 12-week study period, and one reported an escalating situation of intimate partner violence with her boyfriend (#288) which prevented her from being able to seek shelter elsewhere for herself and her child. Participant #263 declined her last counseling session and had difficulty developing an action plan around her distress and concerns for her daughter’s wellbeing.

**Supplemental Figure 1.** Mean SRQ-20 score over study period, by study arm (N=79)^1^


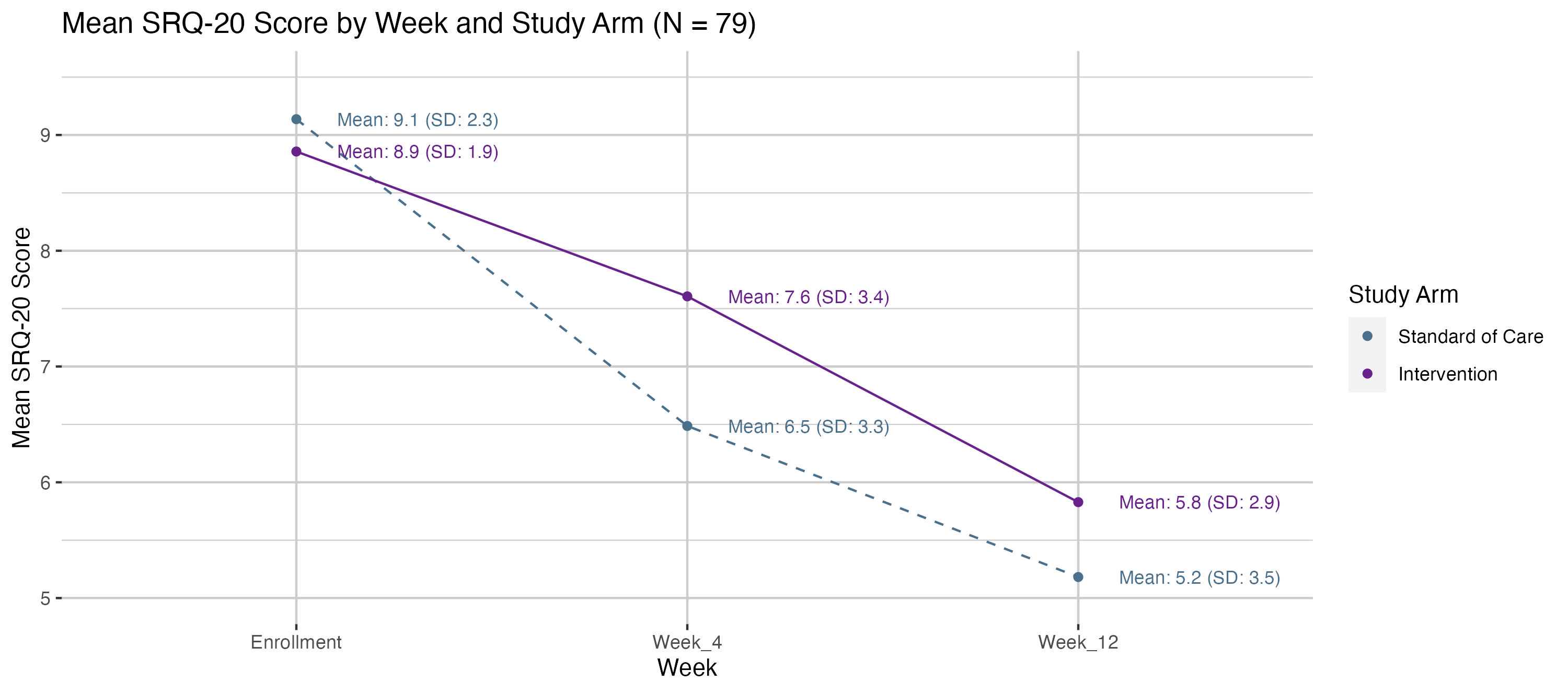


^1^Includes data from the subset of 79 participants retained and with SRQ-20 data available through 12 weeks

**Supplemental Table 1.** PHQ-9 score at Week 12 by randomized arm (N = 79)^1^

| Depressive Symptoms^2^ | **Week 12 Visit** | | | |
| --- | --- | --- | --- | --- |
|  | **Youth Friendship Bench SA** | **Standard-of-care** | **RR (95% CI)^3^** | **p-value** |
|  | *Median PHQ-9 score (IQR)* | |  |  |
| PHQ-9 score, continuous variable^4^ | 5.0 (3.5-9.0) | 5.0 (2.75-9.0) | 0.93 (0.72 – 1.14) | 0.49 |
|  | *Frequency (%)* | |  |  |
| PHQ-9 <10, binary variable^5^ | 26/35 (74.3%) | 35/44 (79.5%) | 0.93 (0.73 – 1.19) | 0.59 |

PHQ-9 = Patient Health Questionnaire; RR = relative risk; 95% CI = 95% Confidence Interval

^1^The per protocol analysis included data from all participants who attended Week 4 and/or Week 12 visits. We restricted the intervention arm sample to those who received a full dose of the intervention, defined as 4-5 individual counseling sessions.

^2^Depressive symptoms were measured using the PHQ-9.

^3^The reference category for all relative risks presented is the standard-of-care group.

^4^PHQ-9 measured as a continuous variable, with a range from 0-27.

^5^PHQ-9 score less than 10 indicates likely no depressive symptoms.

**Supplemental Table 2.** GAD-7 score at Week 12 by randomized arm (N = 79)^1^

| Anxiety Symptoms^2^ | **Week 12 Visit** | | | |
| --- | --- | --- | --- | --- |
|  | **Youth Friendship Bench SA** | **Standard-of-care** | **RR (95% CI)^3^** | **p-value** |
|  | *Median GAD-7 Score (IQR)* | |  |  |
| GAD-7 score, continuous variable^4^ | 5.0 (2.5-10.0) | 5.0 (2.0-9.0) | 1.06 (0.83 – 1.29) | 0.60 |
|  | *Frequency (%)* | |  |  |
| GAD-7 <10, binary variable^5^ | 23/35 (65.7%) | 35/44 (79.5%) | 0.83 (0.62-1.10) | 0.18 |

GAD-7 = Generalized Anxiety Disorder scale; RR = relative risk; 95% CI = 95% Confidence Interval

^1^The per protocol analysis included data from all participants who attended Week 4 and/or Week 12 visits. We restricted the intervention arm sample to those who received a full dose of the intervention, defined as 4-5 individual counseling sessions.

^2^Anxiety symptoms measured through GAD-7.

^3^The reference category for all relative risks presented is the standard-of-care group.

^4^GAD-7 measured as a continuous variable, with a range from 0-21.

^5^GAD-7 score less than 10 indicates likely no anxiety symptoms.

**Supplemental Table 3.** PC-PTSD at Week 12 by randomized arm (N = 79)^1^

| Post-traumatic stress disorder symptoms^2^ | **Week 12 Visit** | | | |
| --- | --- | --- | --- | --- |
|  | **Youth Friendship Bench SA** | **Standard-of-care** | **RR (95% CI)^3^** | **p-value** |
|  | *Median Score (IQR)* | |  |  |
| PC-PTSD sum score, continuous variable^4^ | 2.0 (0-3.0) | 1.0 (0-2.25) | 1.22 (0.80 – 1.84) | 0.35 |
|  | *Frequency (%)* | |  |  |
| PC-PTSD positive screen, binary variable^5^ | 14/35 (40.0%) | 11/44 (25.0%) | 1.60 (0.83 – 3.07) | 0.16 |

PC-PTSD = Primary care post-traumatic stress disorder; RR = relative risk; 95% CI = 95% Confidence Interval

^1^The per protocol analysis included data from all participants who attended Week 4 and/or Week 12 visits. We restricted the intervention arm sample to those who received a full dose of the intervention, defined as 4-5 individual counseling sessions.

^2^Post-traumatic stress symptoms measured through PC-PTSD 4-item checklist.

^3^The reference category for all relative risks presented is the standard-of-care group.

^4^PTSD measured as a continuous count variable, with a range from 0-4.

^5^A screen was considered positive for likely PTSD if participant endorsed at least three items from the PC-PTSD checklist.
